# New Inventions

**Published:** 1898-02

**Authors:** 


### NEW INVENTIONS.

A currycomb, with movable stripper-plates from teeth, held in place with a spring, and readily removable for cleansing.

A horseshoe with removable calks arranged in sockets with set-screws.

An elastic-tread horseshoe, rubber-cushioned in metallic plate, the rubber carrying a stiffening wire, and bolted in the framework.

A concavo-convex, grooved shoe with rubber-cushion in groove.

Elastic-rubber surface for backband of harness.

Combination currycomb and brush, the latter hinged to the comb, to enable it to be used at the same time as the comb.

A reversible animal yoke with a metallic nosepiece.

A nailless horseshoe of a plurality of parts pivotally connected, with insets and a detachable securing-clamp.

A veterinary écraseur, with a cutting and contusing hook-like end, terminating a rod passing through a tubular body-piece, with adjusting-screw at handle for operating.

A metal-armored foot-protector adjusted to pastern and coronet by straps.

An elastic horseshoe composed of rubber, with groove for metal plate, to afford means of attaching to the foot, and rolled for embedding in the rubber.

A pneumatic harness-saddle, pad to retain position when inflated.

A governor or chin-rest for horses, by a spring noseband connected with overdraw checks; chin-strap rings fastened to lever, so that the pressure on the chin-strap and overdraw will be uniform.

An unpadded horse-collar-frame, with pads only on under surface, the frame fastened to saddle by a metal connection, thus keeping throat and neck free from contact.

A portable stall, the posts and partitions having enlargements on ends for adjusting in slots.

An underchecking device, comprising a wire collar, a check-bar, with snap connection, curved jaw rest-pad, nose-straps, and face-pad with snap-hook connection and strap for suspending face-pad from the headstall.

A device for stopping horses, composed of an open steel ring with handle connected with spring, the ring closing over the nose.

